# Food Preferences of the Rubber Plantation Litter Beetle, *Luprops tristis*, a Nuisance Pest in Rubber Tree Plantations

**DOI:** 10.1673/031.009.7201

**Published:** 2009-12-21

**Authors:** Thomas K. Sabu, K.V. Vinod

**Affiliations:** Post Graduate and Research Department of Zoology, St. Joseph's College, Devagiri, Calicut- 673 008, Kerala, India

**Keywords:** *Hevea brasiliensis*, leafage, feeding preference, dormancy, home invasion, Mupli, leaf fall, Kerela

## Abstract

Massive invasion of the litter dwelling beetle, *Luprops tristis* Fabricius (Coleoptera: Tenebrionidae), numbering about 0.5 to 4 million per residential building following summer showers, and their prolonged stay in a state of dormancy, make them an extreme nuisance in rubber tree plantation belts of the Western Ghats in south India. Food preference of post-dormancy adults, larvae and teneral adults stages towards tender, mature and senescent leaves were assessed in three choice and no choice leaf disc tests. All stages have strong preference towards fallen tender leaves and lowest preference towards senescent leaves indicating that leaf age is a major attribute determining food selection and food preference of *L. tristis.* Ready availability of the preferred, prematurely fallen, tender rubber tree leaves as a food resource is suggested as being responsible for the exceptionally high abundance of *L. tristis* in rubber tree plantation belts.

## Introduction

Massive seasonal invasion of the litter dwelling beetles, *Luprops tristis* Fabricius (Coleoptera: Tenebrionidae), following summer showers and their prolonged stay in a state of dormancy (oligopause) is a seasonal nightmare for the farming community in the rubber tree plantation tracts of Kerala along the western slopes of the Western Ghats (Jose 2003; Sabu et al. 2007; Sabu et al. 2008). With their detritivorous habits, harmless effect on the trees, nocturnal surface activity and diurnal passivity in lower litter layers, they would have remained inconspicuous facilitators in litter decomposition and nutrient cycling in monoculture “rubber forests” in the region. However, their massive seasonal invasion into traditional tile roofed residential buildings and thatched sheds, make them an extreme nuisance in rubber belts. Clusters of thousands of invaded beetles crawl inside living rooms and often fall off from ceilings. Subsequently they congregate in dark, undisturbed areas such as attics and wall voids and remain dormant for nine months. Although they do not bite, when disturbed, such as picking them off the walls or when they are squashed or pressed against while sleeping, they release an irritating odoriferous phenolic secretion that causes skin burns. Analysis of seasonality and life cycle of *L. tristis* (Sabu et al. 2008) revealed that larval stages and pre and post-dormancy adults were present in the litter floor only during December to May and virtually no beetles of any stages were found at other times of the year. Perfect synchronism of the oviposition phase of parental (post-dormancy) adults with tender leaf fall at the time of leaf sprouting, and emergence of larval and teneral (pre-dormancy) adult stages with premature fall of leaves were recorded (Sabu and Vinod 2009). Similar instances of premature leaf availability determining the population dynamics of the larvae and neonatal stages of *Chrysophtharta bimaculata* have been reported (Howlett et al. 2001). The suggested link between premature leaf availability with reproductive efficiency of parental adults, survival of early developmental stages in the field and of new generation adults during dormancy (Sabu et al. 2008; Sabu and Vinod 2009), the change in diet of post-dormancy beetles after their return to the field that had previously fed on senescent leaves to feeding on tender leaves with the onset of premature leaf fall in plantations (Sabu et al. 2008), and the feeding preference of larvae and adults on the prematurely fallen wilted leaves (personal observations) implies a relationship between the biology of *L. tristis* and availability of prematurely fallen tender leaves. It raises the question if the high abundance of *L. tristis* in rubber litter stands is related to the availability of prematurely fallen leaves and advantages gained from feeding on fallen tender leaves, then would control of seasonal premature leaf fall in rubber plantations reduce the population build up of beetles? To consider such a possibility, baseline information is required concerning the feeding preferences of the feeding stages (larva, pre-dormancy and post-dormancy adults) of *L. tristis* on different age classes of rubber leaves (tender, mature and senescent) available to *L. tristis* in the litter floor of rubber tree plantations during the period of field presence. The present study provides quantitative evidence in support for the hypotheses that detritivorous *L. tristis* discriminate between fallen tender, mature and senescent leaves and feed preferably on tender leaves.

## Materials and Methods

The present investigation was carried out during January-April 2005 on the Devagiri College campus located 6 km east off the Malabar Coast at Calicut (11° 15′ N, 75° 48′ E), in the Kerala state of India. Three essential components for the study were available, *viz*., an isolated 15 year old rubber tree plantation, RRII 105 clone of *Hevea brasiliensis* Muell. Arg. (Wild.ex ADR. De Jus) (Malpighiales: Euphorbiaceae), a building in the vicinity of the plantation with history of beetle invasion, and a laboratory set up.

Larval and adult food preferences were analyzed with three choice and no choice leaf disk tests. Leaves belonging to three age classes (tender, mature and senescent) were collected from the branches of the same height of a randomly selected rubber tree in the middle of the plantation raised from single clone, to avoid the possible influence of intra-plant variation in leaf quality. Based on phenology studies, freshly sprouted leaves of five days of age were categorized as tender, two weeks following sprouting as mature, and the leaves turning yellowish brown prior to the onset of annual leaf shedding as senescent (Sabu and Vinod 2009). Collected leaves were kept frozen in plastic bags. Fresh leaves were not used as none of the beetle stages fed on fresh leaves of any leaf age class (Sabu et al. 2008). 20 × 20 mm (400 mm^2^) disks of leaves were cut and placed on opposite sides of clay vessel (9 cm diameter × 5 cm height) with thin layer of soil. The vessel was sprinkled with water to moisten the container and soil. Experimental beetles of different developmental stages were introduced into the centre of the vessel and were allowed to feed for 24 h from 8 am to 8 am. Leaf discs were collected, the leaf area consumed by individual beetle and larva from each leaf disc was estimated using a 1 mm^2^ mesh size reticulated paper glued on a glass slide. The amount of leaf disc consumed during the tests was estimated by subtracting the unconsumed area from the initial area of 400 mm^2^. All tests were replicated 30 times.

To ensure uniformity of age at the beginning of the experiment, groups of 60 teneral adults and 3^rd^ instar larvae in the premoulting quiescence stage were collected from plantation litter and post-dormancy beetles in the refractory phase were collected from their natural aggregation sites in the college hostel premises. Fourth instar larvae were not sufficiently abundant on same day so the tests had to be conducted at two occasions on successive days. Teneral adults and post-dormancy adults that had emerged on a single day were used. All stages were reared in clay vessels placed in an environmental chamber and fed with diced leaves of all three types for four days to reduce the effect of leaf quality variations on growth rate. Each stage was deprived of food for 12 h before the starting of the experiments. Feeding experiments on post-dormancy adults were conducted during the 3rd week of January, while tests on larvae and teneral adults were conducted during the 2^nd^ week of March. Three choice and no choice experiments were conducted on successive days employing frozen leaves.

Significance levels of variation in food consumption by each life cycle stage (larva, pre-dormancy and post-dormancy adults) on leaf types (tender, mature and senescent leaves) were analyzed with two-way ANOVA in both three choice and no choice conditions. Variations in the quantity of each leaf type consumed by each life cycle stage between three choice and no choice conditions were analyzed with t-test (Gujarati 2003). Megastat, version 10.0 (Orris 2005) was used for all statistical analysis.

## Results

All stages *viz.*, larva, pre-dormancy and post-dormancy adults, preferred tender leaves over mature and senescent leaves. Senescent leaf was the least preferred food in three choices as well as in no choice experiment (Table 1).

Significant differences in the quantity consumed by three life cycle stages among leaf types as well as in the rate of consumption among three life cycle stages in both tests were distinct (three choice test- among leaf age classes *F_2_* = 363.23, *P* <0.01, among life cycle stages *F_2_* = 45.26, *P* <0.01; no choice test- among leaf age classes *F_2_* = 209.87, *P* <0.01, among life cycle stages *F_2_* = 5.81, *P* <0.01). Among three stages, pre-dormancy adults consumed more food than post-dormancy adults or larva and larva consumed the least.

**Table 1.**
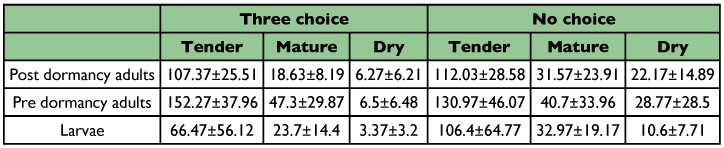
Quantity of leaves consumed (mean±SD of 30 replicates) by three life cycle stages of *L. tristis* under three choice and no choice experimenttests. All values are in mm^2^.

Post-dormancy adults did not show significant differences in consumption of tender leaf in no choice tests than in three choice tests (t_57_ = -0.67, >0.05) . However, with mature (t_35_ = -2.8, *P* <0.05) and senescent leaves (t_38_ = -5.4, *P* <0.05), there was significant variation in consumption. Predormancy adults did not show any significant differences in the consumption of tender (t55 = 1.95, *P* >0.05) and mature (t_57_ = 0.8, *P* >0.05) leaves, while they consumed more senescent leaves in no choice tests (t_31_ = -4.17, *P* <0.05). Larval stages consumed more of each leaf age class in no choice tests (t_56_ = -2.55, *P* <0.05, t_53_ = -2.12, *P* <0.05, t_39_ = -4.72, *P* <0.05 in tender, mature and senescent leaf age classes respectively).

## Discussion

All stages of *L. tristis* showed significant preference for wilted tender leaves and lowest preference for senescent leaves, highlighting the importance of leaf age in determining the food selection and food preference and also the existence of preference hierarchies. High nutritional value (variations in host quality) for unknown reasons could explain the high preference towards tender leaves (preference hierarchies) (Coley 1980; Ernest 1989; Bernays and Chapman 1994; West and Cunningham 2002). However, feeding on mature and senescent leaves, even when tender leaves were available during three choice tests, logically raises questions about the possible reasons for feeding on mixed diets and the advantages it provides. The importance of host quality variation in the establishment of preference hierarchies and host plant selection by phytophagous insects (Rausher 1981; Papaj 1986; Thompson and Pellmyr 1991; West and Cunningham 2002) and non-availability of tender leaves to post dormancy adults (Sabu et al. 2008; Sabu and Vinod 2009), suggest that feeding on mixed diets by all stages must be a learned adaptive strategy of *L. tristis* to avoid overspecialization on nutritionally superior but highly seasonal (i.e. limited) tender leaves. Specialization of *L. tristis* on highly seasonal tender leaves would have been detrimental to the fitness of all stages, more specifically for post-dormancy adults returning to the plantation litter at a time when only senescent leaves are available. Though leaves of all the age classes are available in plantations when larvae and teneral adults emerge, over specialization on tender leaves would have affected their survival chances if by any reason prematurely fallen tender leaves are not available to feed upon. This is another instance of establishment of preference hierarchies in the feeding behaviour of phytophagous insects in the face of host limitation (Courtney et al. 1989; Thompson and Pellmyr 1991; Bernays and Chapman 1994; Mayhew 1997). Avoidance of overspecialization on tender leaves enables switching over between the more readily available but nutritionally inferior senescent leaves and seasonal but nutritionally superior tender leaves, irrespective of their innate preferences towards the former. Since the tests employed the food that all beetle stages were found feeding on the plantation litter, and the stages were field collected rather than laboratory raised, suggests that these food preferences reflect their natural feeding behaviour.

Among the three stages, the pre-dormancy (teneral adults) stage is the most aggressive feeder of each leaf type and it may appear that during the preparatory phase of dormancy they are stocking up on energy resources (Denlinger 1986; Leather et al. 1995). However, dormancy in *L. tristis* is oligopause, which does not involve long term advance preparations by accumulation of energy reserves prior to active dormancy (Mansingh 1971; Sabu et al. 2008). Therefore, the aggressive feeding of teneral adults might be an instance of intensive feeding related to the developmental requirements of young adults. Long lived adults with more feeding time accumulate more energy resources and have lesser mortality during dormancy in comparison to the younger adults with shorter feeding time. Earlier studies (Sabu et al. 2008) showed that pre-dormancy adults have an abdomen full of reserve materials when they enter dormancy; post-dormancy beetles on their return to the field go on a feeding spree lasting two weeks prior to breeding activities; the larval phase starts when premature leaf fall is midway through.

Preference of all stages towards tender leaves and the synchronicity of premature leaf fall and the field presence of all three stages indicate that premature leaf fall in rubber plantations contributes to the unprecedented abundance of *L. tristis* beetles, which is uncommon in other natural or monoculture litter stands and it raises the following practical questions. What are the nutritional reasons behind the predisposition towards tender leaves? If tender leaves by way of premature fall of leaves are not available during the period, how will it affect the reproductive capacity of post-dormancy adults, duration of the larval phase, quality and quantity of reserve food accumulated by the teneral adults and the survivability of teneral adults during the dormancy phase? If the high abundance of *L. tristis* in rubber estates, compared to their very low abundance in natural forests, is related to the advantages gained from feeding on fallen tender leaves, then will not the control of seasonal premature leaf fall in rubber plantations cause by the powdery mildew, *Odium hevea*, and *Corynespora* leaf disease, *Corynespora cassiicola* Jacob 2002) will enable the control of this menace? These questions become relevant in view of the observation that although the Rubber Board, Govt. of Kerela recommends various measures to control the premature fall during the leaf sprouting period, our interactions with the planters across the region revealed that this is not practiced as the high labor costs outweigh the benefits.
